# Editorial: Neuro-motor control and feed-forward models of locomotion in humans

**DOI:** 10.3389/fnhum.2015.00306

**Published:** 2015-06-02

**Authors:** Marco Iosa, Leonardo Gizzi, Federica Tamburella, Nadia Dominici

**Affiliations:** ^1^Clinical Laboratory of Experimental Neurorehabilitation, Fondazione Santa Lucia I.R.C.C.S.Rome, Italy; ^2^Department of Neurorehabilitation Engineering, Bernstein Focus Neurotechnology Göttingen - Bernstein Center for Computational NeuroscienceGöttingen, Germany; ^3^SPInal REhabilitation Lab (SPIRE), Fondazione Santa Lucia I.R.C.C.S.Rome, Italy; ^4^Faculty of Human Movement Sciences, MOVE Research Institute, VU University AmsterdamAmsterdam, Netherlands

**Keywords:** gait, walking, central pattern generators (CPG), motor control, neurorehabilitation

“He told me with amusement that when one is walking rapidly each step takes no more than half a second, and in that half second no fewer than 54 muscles are set in motion. I listened in awe. I at once directed my attention to my legs and tried to discover the infernal machine. I thought I had succeeded in finding it. I could not of course distinguish all its 54 parts, but I discovered something terrifically complicated which seemed to get out of order the instant I began thinking about it.”

Well-depicted by Svevo in “Confessions of Zeno” (Svevo, 1923, 1989), the act of walking involves many different muscles and the necessity of controlling several degrees of freedom at once. This Research Topic has mainly been focused on the strategies adopted by the central nervous system for reducing the complexity of motor control and compensating for the sensorimotor delays. The studies published within this Research Topic addressed this issue at two levels of investigation, focusing on one side the neural circuitry, such as the so called central pattern generators in the spinal cord and the supraspinal structures, and on the other one on the cognitive processes involved during locomotion.

One of the paramount discoveries in locomotion is the existence of a central pattern generator (CPG), i.e., a neural circuitry within the spinal cord that can autonomously generate basic locomotor rhythmic patterns, even in the absence of brain connections and sensory information (Grillner, 1985). Although there is compelling evidence of existence of CPG in humans, a final proof is still lacking, also because CPGs have generally been investigated in reduced models including *in vitro* isolated preparations, genetically-engineered mice, spinal cord-transected animals, and virtual models. Guertin (2014) presented an extensive review of studies, concluding that the development of CPG-modulating clinical therapies is a necessary step for improving the locomotor function in patients with spinal cord injury. Similarly, the study of Dzeladini et al. (2014) enters in the debate between CPG and reflex-based human neuro-musculo-skeletal models, supplying a mixed model in which CPG are integrated in a reflex-based model. Their results highlighted potential advantages of CPGs as feed-forward components that can be interpreted as feedback predictors for stabilizing gait modulation. Further, their model perfectly replicated the harmonic structure of human gait (that has recently been found based on the so called golden ratio Iosa et al., 2013). Golden ratio is an irrational number at the basis of many biological and physical systems showing a omotetic harmonic structure, and the ratio between durations of stance and swing phases was found to coincide with the golden ratio (Iosa et al., 2013).

The hypothesis of Dzeladini could be supported by the results of two other studies published in this Research Topic. Awai and Curt (2014) reported a loss of intralimb coordination, especially related to the inability of modulating coordination when increasing speed from slow to comfortable, in patients with spinal cord injury. La Scaleia et al. (2014) found that coordination may be based on a discrete, temporal harmonic cyclic structure, along which, critical points delimiting burst components are shifted. In particular, despite the differences in the segmental level and intensity of the spinal activity, the motor-neurons' activation patterns exhibited two major bursts during different locomotor tasks: one around heel strike and the other around toe off, again in line with a schema of activations strictly related to the harmonic structure of gait. Practical guidelines on the methodological aspects for extracting neural control information in the guise of motor modules through electromyographic muscle activation patterns have been clearly depicted in the study of Oliveira et al. (2014).

Patients with complete sensory-motor lesions have a very limited chance of recovering the ability of walking and even if they recover the ability to ambulate, they are usually limited ambulators. The chances of walking recovery improve in less severe lesions and younger age. Motor and somatosensory evoked potentials can contribute toward diagnosing lesions of different neural structures and predicting the recovery of functional movements, as reported in the review by Scivoletto et al. (2014). The same group contributed also with an interesting study on the effects of enhancing somatosensory inputs through the application of kinesio-taping: spasticity can be reduced and gait ability improved in patients with spinal cord injury (Tamburella et al., 2014). These results are hence in line with the above reported importance of sensory feedback for modulating the rhythmic activity of motoneurons activations (La Scaleia et al., 2014). As described by Scivoletto et al. (2014), during the long and strenuous neurorehabilitation of patients with spinal cord injury, learning-dependent changes in CPG circuits can occur primarily through rhythmic peripheral influences imposed by the exercises. The gait training for these patients can also be improved through visual biofeedback, as reported by Schlieβmann et al. (2014), or robotic devices as reported in the studies of Del-Ama et al. (2014) and Sylos-Labini et al. (2014). In the former study the muscle examination of patients with spinal cord injury revealed improvements at knee and hip sagittal muscle functioning, the same joints as those found impaired in the study of Awai and Curt (2014). The latter reports the gait of subjects with spinal cord injury using a specifically developed wearable ambulatory exoskeleton (Sylos-Labini et al., 2014).

A comparison of the effects of robotic therapy against those obtained with sensory-based training in subjects with multiple sclerosis is reported in the study of Gandolfi et al. (2014). Multiple sclerosis is a chronic disease of the central nervous system characterized by a progressive decline in various neurological functions, with locomotion disturbances primary related to a reorganization of the postural control system and to deficits of central integration of sensory afferents. Outcomes resulted similar after the two different therapies, with a more pronounced improvement in gait function and balance, for robotic-aided and sensory integration based training, respectively.

The benefits obtained by gait training based on enhancing sensory feedback reported in the above studies suggest that human gait may involve a complex interplay between spinal and cortical circuits.

In fact, not only spinal, but also supraspinal structures are involved during locomotion. It has been shown that they are responsible for initiating (Jiang et al., 2015) and modifying the features of the gait basic rhythm, for stabilizing the upright walking, and for coordinating movements in a dynamic changing environment (Grasso et al., 2004). Furthermore, specific damages of supraspinal structures result in specific alterations of human locomotion, as evident in subjects with brain injuries such as stroke (Clark et al., 2010; Gizzi et al., 2011), brain trauma, or people with cerebral palsy (Iosa et al., 2012), in people with death of dopaminergic neurons in the *substantia nigra* due to Parkinson's disease, or in subjects with cerebellar dysfunctions, such as ataxia (Kirtley, 2006). The role of cerebellum during locomotion has been shown to be related to the coordination and adaptation of movements. Cerebellum is the structure of CNS where the internal models—neural representations miming meaningful aspects of our body, such as input/output characteristics of sensorimotor system—are conceivably developed (Wolpert et al., 1998). Internal model control has been shown to be at the basis of motor strategies for compensating delays or lack in sensorimotor feedback. Some aspects of locomotion require predictive internal control, especially for improving gait dynamic stability, avoiding obstacles, or when sensory feedback is altered or compromised. In their review focused on cerebellar contribution to feed-forward control of locomotion, Pisotta and Molinari (2014) hypothesized that sequence recognition is the mechanism by which the cerebellum facilitates the control of gait. Once again, the repetition of specific events during locomotion embedded into a predictable sequence seems to be a key-factor for facilitating locomotor control.

Sale et al. (2014) showed that, as for subjects with spinal cord injury, repetitive robotic gait training resulted effective also in a group of subjects with progressive supranuclear palsy, a rare neurodegenerative disease that causes the gradual deterioration and death of specific volumes of the brain (in particular midbrain, pallidum, thalamus, subthalamic nucleus, frontal lobes).

The role of cognitive functions during locomotion is still debated, with some authors (e.g., Ruchinskas et al., 2000) suggesting that locomotion is a largely automatized action, and others (Lamoth et al., 2011), who found that stability of gait is altered when cognition is impaired or during dual tasking in frail healthy people. The study of Meester et al. (2014), showed a greater activity in the prefrontal cortex, when a cognitive load was administered to the subject during walking. This adaptation, however, did not detrimentally affect the amplitude of soleus H-reflex or the spatiotemporal variables of gait. Analogously no correlation between walking speed and prefrontal cortex activity was found. On the other hand, the study of Begg et al. (2014) showed how other aspects of walking (e.g., increasing minimum toe clearance in subjects at risk of fall) can be improved through cognitive-motor training, such as a visual biofeedback.

The role of vision in gait is probably the most evident aspect needing the involvement of cortical areas during walking in the surrounding environment. Aprile et al. (2014) found that subjects with strabismus adopt different walking strategies to compensate their deficits. They found that subjects with exotropia (an expanded visual field), showed larger step width than subjects with esotropia (a reduced visual field), suggesting a specific neurosensorial adaptation of gait with respect to abnormal binocular cooperation. These results are in line with the famous quote “Go where I'm looking, not look where I'm going” by Berthoz in his famous book “The brain's sense of movement,” claiming the role of gaze-based feed-forward control involved in locomotion along a desired trajectory (Berthoz, 2000). The title of the study of Iosa and colleagues published in this Research Topic, “The brain's sense of walking… ” (Iosa et al., 2014), is a clear tribute to Berthoz's work. In that study, as in the one from Fusco et al. (2014), the ability of imagining walking was under investigation. Motor imagery has been deeply investigated in literature, and it has been defined as a mental representation of an action without its physical execution. Fusco et al. (2014) pointed out three aspects about the intertwine between motor imagery and motor execution of gait actions: (1) they are correlated, but not always coincident; (2) agreement occurred only for some specific usual locomotor tasks (such as forward walking, but not for example for lateral walking); (3) motor execution resulted better simulated during dynamic motor imagery, than during static motor imagery, i.e., when a movement simulating the real one was performed. The study of Iosa et al. (2014) added that dynamic locomotor imagery is less formed in children with typical development and is impaired in children with cerebral palsy.

The abovementioned studies pointed out a number of neural structures involved in locomotion, which seem to paradoxically complicate, instead of simplifying, the management of all sensory and actuator systems necessary for the harmonious execution of human locomotion. However, the results published in this Research Topic appear to converge toward an intrinsic simplification of the problem: the involved neural systems seem to be responsive to the repetitive sequences of events occurring during gait, being facilitated in the generation, control, and prediction of walking by its intrinsic harmonic structure. Despite further studies being needed, neuroscience is giving important suggestions for a more effective neurorehabilitation, and for answering the question that arises when observing the elegant coordination and interplay of movement and balance, joints and muscles, senses and actuation, involved in human walking.

## Conflict of interest statement

The authors declare that the research was conducted in the absence of any commercial or financial relationships that could be construed as a potential conflict of interest.
